# Enhancing Mechanical Properties of the Spark Plasma Sintered Inconel 718 Alloy by Controlling the Nano-Scale Precipitations

**DOI:** 10.3390/ma12203336

**Published:** 2019-10-13

**Authors:** Shuaijiang Yan, Yun Wang, Qingxiang Wang, Chengsong Zhang, Dazhi Chen, Guodong Cui

**Affiliations:** 1 School of Materials Science and Engineering, Southwest Jiaotong University, Chengdu 610031, China; ysj@my.swjtu.edu.cn (S.Y.); wy_swjtu@163.com (Y.W.); wangqx1981@163.com (Q.W.); cszhang@home.swjtu.edu.cn (C.Z.); cdz@swjtu.edu.cn (D.C.); 2 Sino-Euro Materials Technologies of Xi’an Co., Ltd., Xi’an 710018, China; 3 Key Laboratory of Advanced Technologies of Materials, Ministry of Education, Chengdu 610031, China

**Keywords:** Inconel 718 superalloy, spark plasma sintering, nano-scale precipitation, microstructure, mechanical property

## Abstract

The present study aimed to optimize the phase constituents and mechanical properties of the spark plasma sintered (SPS) Inconel 718 (IN718) alloy. A series of heat treatment routes were designed based on the phase relations in IN718 and performed for the optimization. The microstructure and phase compositions of the SPS IN718 alloys were examined by using X-ray diffraction (XRD), scanning electron microscopy (SEM), energy disperse spectroscopy (EDS), and transmission electron microscopy (TEM). The mechanical properties of the samples were characterized at room temperature and at 650 °C. The results showed that large amounts of γ” (Ni_3_Nb) and γ’ (Ni_3_(Al, Ti)) strengthening phases precipitated in the IN718 alloy after direct aging (DA) of the as-fabricated sample. Moreover, the mechanical properties of the DA sample were comparable to that of the best one of the solution-treated and aged counterparts. The analysis showed that the rapid sintering and solid solution treatment of the IN718 alloy were achieved simultaneously by SPS. In the case of the SPS IN718 material, the direct aging regime had the same heat treatment effect as the conventional solid solution and aging treatment. This contributes toward improving the production efficiency and reduces manufacturing costs in the actual production process.

## 1. Introduction 

As a precipitation strengthening alloy, the Inconel 718 (IN718) alloy has been widely used in the aircraft industry due to its good corrosion resistance and excellent mechanical properties at elevated temperatures [1,2,3]. IN718 is strengthened mainly by the nano-scale γ” (Ni_3_Nb) and γ’ (Ni_3_(Al, Ti)) phases. The γ” phase has a disc shape with a body centered tetragonal (bct) structure [4,5]. The γ’ phase has a cubic (fcc) structure and precipitates as a cube-to-cube relationship with the matrix [6]. Due to the composition of the alloy, there will be more γ” than γ’ in the alloy. The γ” phase is metastable and can transform into the δ (Ni_3_Nb) phase at high temperatures [7,8,9]. Appropriate amounts of δ phases at grain boundaries can refine the grain sizes and improve the resistance to grain boundary creep fracture [10,11,12]. However, large amounts of δ phases will decrease the mechanical strength, since it consumes Nb atoms, leading to the loss of γ” phases in this alloy [13]. The relatively high Nb concentration also results in the precipitation of carbide (NbC) and Laves ((Ni, Fe, Cr)_2_(Nb, Mo, Ti)) phases in IN718.

With the development of high strength, precision, and fabrication efficiency of IN718 components, the conventional casting and forging methods are difficult to meet the increasingly strict requirements [14]. Spark plasma sintering (SPS), as a relatively new powder metallurgy (P/M) technique, is well-suited for the preparation of IN718 alloy due to its assurance of high fabrication efficiency, relative density, and compressive strength of the SPS IN718 material [15]. Compared with other P/M techniques, SPS is assisted by a highly-pulsed DC, which provides the heat directly inside the sample by the Joule effect [16]. With the simultaneous application of uniaxial pressure, the samples can be fabricated rapidly by SPS [17]. Due to these unique characteristics of the SPS technique, the microstructure of the SPS material is markedly different from that of the same material fabricated by other P/M methods [15,18,19,20]. The special microstructures of SPS materials will require different post-process heat treatment in order to obtain the desired properties. Niu et al. [21] reported that the solution annealing temperature required for the SPS Ti-22Al-25Nb alloy is lower than the SLMed product. The work of Chua et al. [22] and Mahathaninwong et al. [23] indicated that the similar heat treatment route had various effects on the mechanical properties of SPS and rheocasting 7075 alloys. Mondet et al. [24] found that for the SPS-fabricated AZ91 alloy, the in-situ precipitation treatment is more preferred than the traditional T6 heat treatment because it led to a more significant improvement of the mechanical properties. Therefore, the heat treatment regime of the SPS-fabricated alloys needs to be optimized because of their special microstructures. As for the SPS-fabricated IN718, existing studies adopted the heat treatment schedule for the cast and wrought materials [15,25]. The traditional solution treatment and aging scheme shows the strengthening effects on SPS IN718. However, the optimized heat treatment route specifically for SPS IN718 alloy remains unclear, and methods for tuning the mechanical properties through heat treatment are short of guidance. Therefore, it is of upmost importance to design the specific heat treatment route for the SPS IN718 alloy with the aim of tailoring the microstructure and tuning the mechanical properties.

In this study, a series of heat treatment routes were designed based on the phase relations in IN718 and performed on the SPS-fabricated IN718 alloy. The mechanical properties of samples with different heat treatment were evaluated, and the results were related to the phase characterization. The microstructural evolution of the SPS IN718 alloy was also studied within the steps of sintering and heat treatment. It appears that the SPS IN718 shows a distinctive microstructure and its strength can be tuned by post-process heat treatment.

## 2. Experimental Procedures

**Figure 1:**
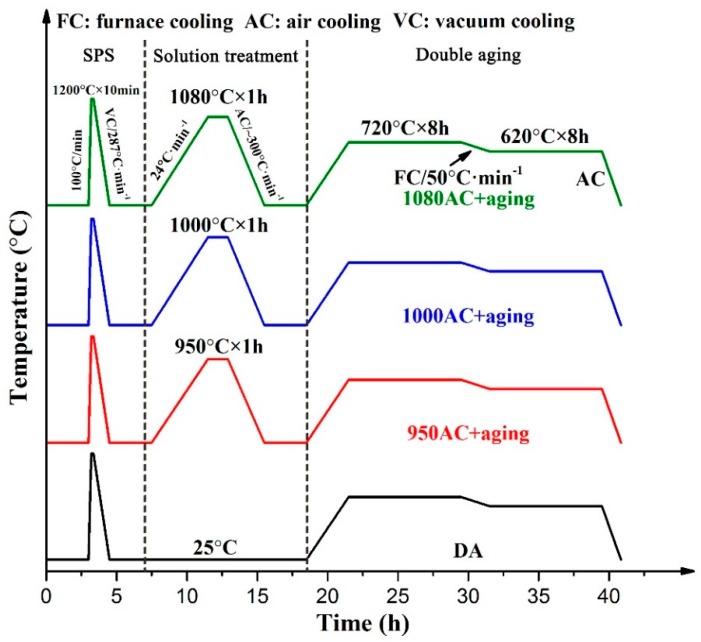
Schematic illustrations of spark plasma sintering (SPS) and the heat treatment cycles performed in this study.

The Inconel 718 powder (Sino-Euro Materials Technologies of Xi’an Co., Ltd., Xi’an, China) manufactured by the plasma rotation electrode process (PREP) was used as the starting material. The Inconel 718 powders are spherical with a diameter of 15–53 μm. The elemental composition of the powder was determined by inductive coupled plasma (ICP) analysis, as presented in Table 1. The Inconel 718 alloy was fabricated using a SPS system (model HD-10 from FCT Germany, Gewerbepark, Germany) at a temperature of 1200 °C, soaking for 10 min under 51 MPa pressure. The detailed sintering parameters can be seen in our previous work [15]. The post-process heat treatments were conducted according to the following routes: (1) direct aging (720 °C, 8 h/furnace cooling at 50 °C/h to 620 °C, 8 h/air cooling) (designated as DA); (2) solution treatment conducted at 950 °C (1 h/air cooling) + double aging (720 °C, 8 h/furnace cooling at 50 °C/h to 620 °C, 8 h/air cooling) according to the AMS 5663 standards [26] for wrought IN718 (designated as 950AC+aging); (3) solution treatment conducted at 1000 °C (1 h/air cooling) + double aging (same as the 950AC+aging samples) (designated as 1000AC+aging); and (4) solution treatment conducted at 1080 °C (1 h/air cooling) + double aging (same as the 950AC+aging samples; designated as 1080AC+aging). The schematic illustrations of SPS and heat treatment cycles are shown in Figure 1.

For observing and analyzing the microstructures, samples were ground by SiC paper and polished with a 3 μm diamond suspension. The Kalling reagent (100 mL ethanol, 100 mL hydrochloric acid, and 5 g copper chloride) was used as the etching solution. A scanning electron microscope (SEM, JSM-6900F, JEOL, Tokyo, Japan), equipped with energy dispersive X-ray spectroscopy (EDS) was used to complete the microstructure characterization of the IN718 powder and fabricated alloys. Thin foil samples were prepared by mechanical thinning followed by ion milling at room temperature and were analyzed by the transmission electron microscope (TEM, JEM-2100, Tokyo, Japan). Phase constituents of the IN718 samples were analyzed based on the X-ray diffraction (XRD, X’ pert pro, PANalytical B. V., Almelo, the Netherlands) results. The Cu K-alpha radiation was used, and the Bragg angles were selected from 30° to 80° with a scanning rate of 5° per minute.

The average Vickers hardness values were obtained with at least ten indentations. A micro hardness tester (HVT-1000, Lanzhou Zhongke Kaihua Technology Development Co., Ltd., Lanzhou, China) was used to complete the hardness tests at both room temperature and 650 °C. For compressive tests, cylindrical samples with a diameter of 8 mm and height of 12 mm, as is suggested in the ASTM E9-89a (2000) standard [27], were prepared by the electro-discharging machine. Compressive tests were conducted using an electrical universal material testing machine (WDW-200, Changchun Kexin Test Instrument Co., Ltd., Changchun, China) at a strain rate of 1.1 × 10^−3^ s^−1^, and at least three samples were tested for each condition. 

## 3. Results 

### 3.1. Microstructure of the IN718 Powder and the SPS IN718 Alloys

**Figure 2:**
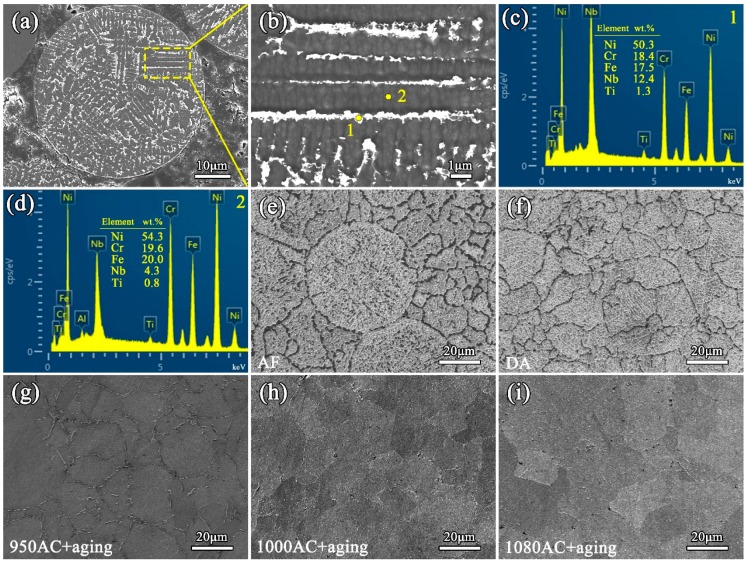
(**a**) Cross-sectional morphology of the IN718 powder. (**b**) The enlarged view of the powder microstructure in the region marked in (**a**). (**c**,**d**) Energy disperse spectroscopy (EDS) spectra and elemental contents of location 1 and 2 (marked in (**b**)), respectively. (**e**–**i**) Microstructures of the as-fabricated, direct aging (DA), 950AC+aging, 1000AC+aging, and 1080AC+aging samples.

**Figure 3:**
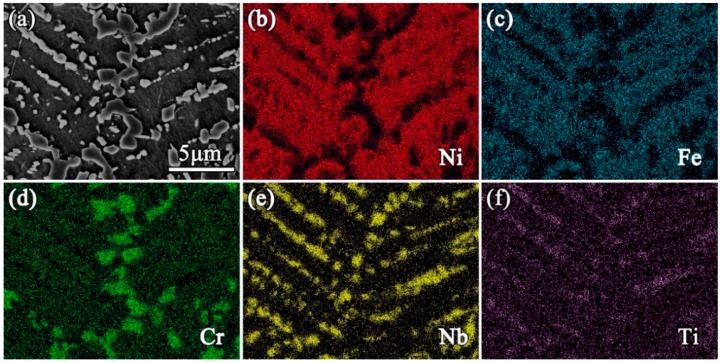
EDS elemental distribution of the as-fabricated sample: (**a**) SEM image of the tested field, (**b**) Ni, (**c**) Fe, (**d**) Cr, (**e**) Nb, (**f**) Ti.

Figure 2 shows the cross-sectional morphology of the IN718 powder and microstructures of the SPS IN718 alloys. The microstructure of the IN718 powder (see Figure 2a) showed the dendritic grains, which was caused by the solidification partition coefficient difference of element compositions during the rapid cooling step of powder manufacturing. The powder EDS results, shown in Figure 2c,d, indicate that Nb and Ti segregated more severely in the interdendritic region than at the center of the dendrites. Meanwhile, Ni and Fe were more prone to aggregating in the dendrite cores. The microstructure of the as-fabricated (AF) samples (see Figure 2e) contained some block-shaped and bar-shaped segregation. It should be noted that the segregation constitutes a dendritic microstructure, although the outline of the dendritic grains was discontinuous (as also can be seen in Figure 3a). In order to confirm the element types in the segregation, the elemental map of EDS testing was conducted on the as-fabricated samples. As shown in Figure 3, Ni and Fe mainly segregated in the dendrite arms, while Nb and Ti segregated more severely in the interdendritic region. Moreover, the Cr element segregated in both block-shaped segregation and the dendrite arms. For the DA samples (see Figure 2f), the micro-segregations were not totally removed, because of the lack of the solution treatment. As shown in Figure 2g–i, the microstructures of the solution-treated samples became more uniform, indicating that the elements diffused evenly into the matrix after solution treatment. The grain sizes of the SPS IN718 were measured by the Heyn linear intercept procedure in no less than three fields for each sample according to the ASTM E112 standard [28]. For the AF, DA, 950AC+aging, 1000AC+aging, and 1080AC+aging samples, the average grain sizes were ~10 μm, ~9.1 μm, ~11 μm, ~13.8 μm, and ~20.4 μm, respectively. It should be noted that the average grain size of 1080AC+aging samples was much larger than that of other samples. 

**Figure 4:**
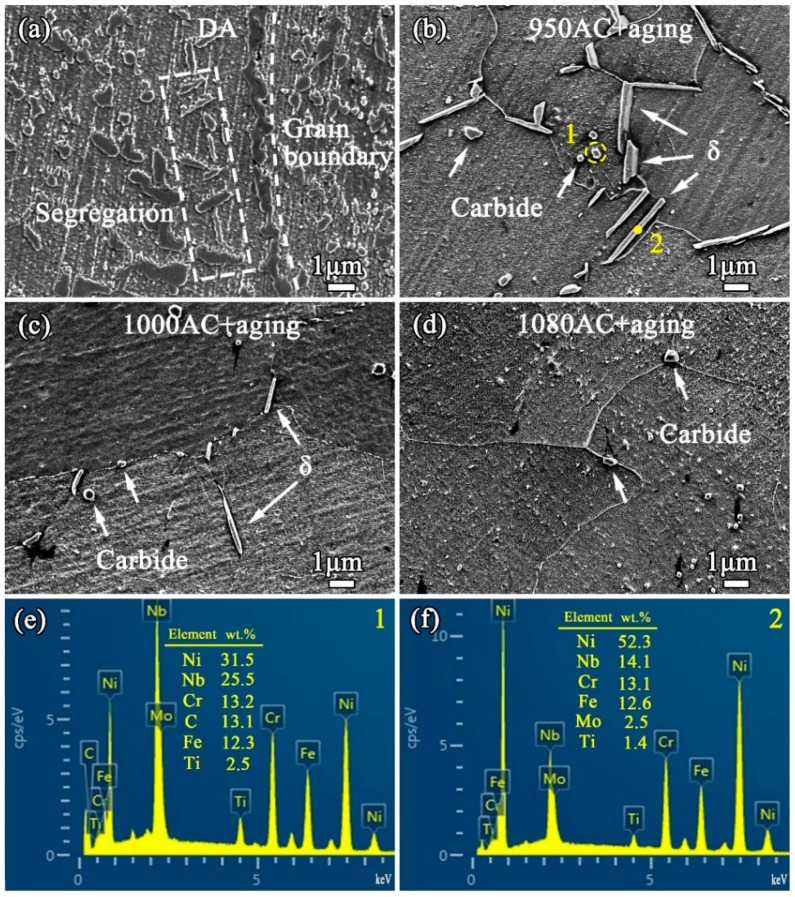
High-magnification scanning electron microscopy (SEM) micrographs of (**a**) DA, (**b**) 950AC+aging, (**c**) 1000AC+aging, and (**d**) 1080AC+aging samples. (**e**) and (**f**) are EDS spectra and elemental contents of the carbide and δ phases.

Figure 4 presents the high-magnification SEM images of the SPS IN718 alloys. In the case of DA (see Figure 4a), the element segregation was inherited from the AF sample. As can be observed from the 950AC+aging samples (see Figure 4b), some minor phases precipitated along the grain boundaries. EDS analysis was carried out to identify these phases. Two types of precipitates were observed, the first one was block-shaped and rich in Nb and C (Figure 4e) and identified as the MC-carbide phases. The carbides mainly precipitated at grain boundaries, but could also be found in the grain interiors. The second type were precipitates that have a needle shape and are rich in Ni and Nb (see Figure 4f), identified as the δ-Ni_3_Nb phase according to the phase characteristics of IN718 [29]. For the 1080AC+aging samples, the grain boundaries were free of the δ phase (Figure 4d) because the 1080 °C exceeded the solvus of δ phase. However, the solution treatment temperatures in the present study were lower than the solvus of NbC (around 1250 °C [30]), and the carbide phases could not be totally removed from the microstructure.

### 3.2. XRD Analysis 

**Figure 5:**
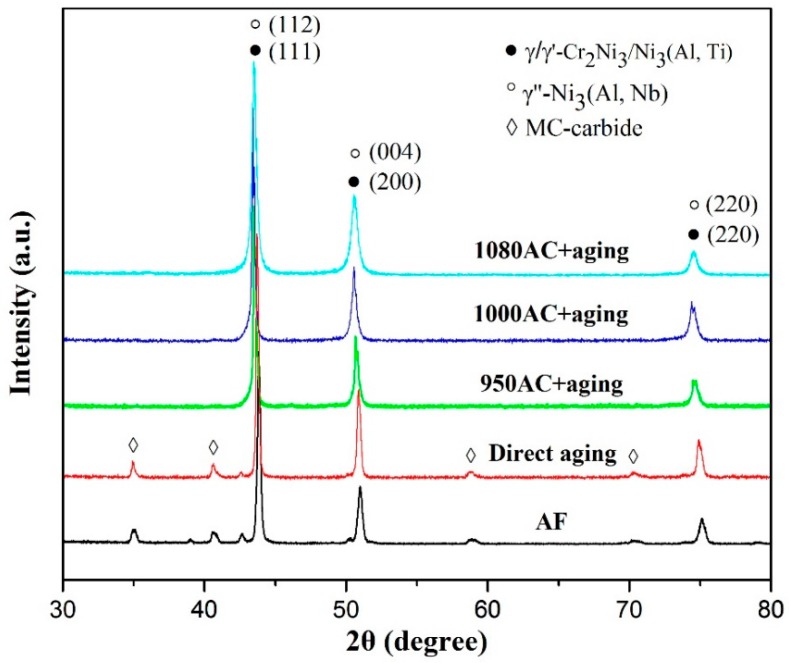
The X-ray diffraction (XRD) patterns of as-fabricated and heat-treated IN718 alloys.

The X-ray diffraction (XRD) patterns of the as-fabricated and heat-treated IN718 alloys are presented in Figure 5. The diffraction peaks of γ’ and γ” phases could not be well distinguished because they overlapped with the peaks of the γ matrix. The peaks of the carbide phase could be observed in the XRD results of the as-fabricated and directly aged samples. For the solution-treated and aged samples, the XRD patterns were similar to the DA and AF samples; however, the diffraction peaks of γ matrix shifted to the lower angles. The XRD peak shifting may have been caused by the growing of the lattice parameters of the γ-matrix. During solution treatment, the elements such as Nb and Ti could be dissolved into the γ-austenite lattice. These foreign atoms generally existed in the interstitials of the γ-FCC lattice [31], causing the enlarging of the γ lattice and leading to the XRD peak shifting. Because Nb and Ti are also the main carbide formation elements, the intensity of the XRD peaks representing the carbide phase became lower in the solution-treated samples.

**Figure 6:**
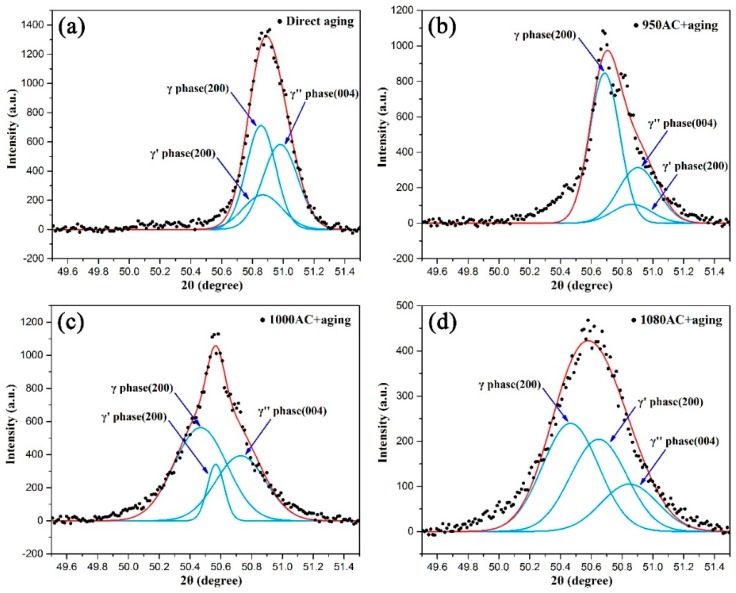
The powder X-ray diffraction (PXRD) patterns and corresponding Gaussian fitting results for the (**a**) DA, (**b**) 950AC+aging, (**c**) 1000AC+aging, and (**d**) 1080AC+aging samples.

As shown in the XRD patterns presented in Figure 5, the diffraction peaks of γ, γ’, and γ” phases could hardly be distinguished because the positions of diffraction peaks on the (111), (200), and (220) planes of γ/γ’ phases were close to those on the (112), (004), and (220) planes of γ”. As a result, the precipitation behavior of γ’’ and γ’ phases needed to be analyzed by peak separation of the lower angle diffraction peaks on the (200) plane of γ, (200) plane of γ’, and (004) plane of γ’’. The peak separation software, PeakFit for Windows [32], was used to complete the PXRD analysis by applying Gaussian fitting. The corresponding fitting results are shown in Figure 6. Based on the integral area of the separated peaks, the proportion of γ” phase in the DA, 950AC+aging, 1000AC+aging, and 1080AC+aging samples was determined to be 40.4%, 29.4%, 35.6%, and 19.1%, respectively, while the contents of γ’ phase were 18.3%, 10.0%, 10.4%, and 37.2%. According to the fitting results, the number of γ” phase increased when solution-treated at 1000 °C and then dropped at 1080 °C, whereas γ’ phase precipitated at higher solution treatment temperatures. Moreover, γ” and γʹ phases presented their maximum precipitation tendencies in the DA and 1080AC+aging samples, respectively.

The lattice parameters and misfit values calculated from the fitting results are listed in Table 2. For the SPS-fabricated IN718, the lattice parameters of the γ-matrix were larger than those of the γ’ and γ” precipitates. The lattice parameters of the γ-matrix increased with the elevation of solution treatment temperatures because more alloying elements diffused into the γ lattice at higher temperatures, causing the lattice expansion. The lattice misfit is used to evaluate the strain state at the interfaces of phases, which greatly influences the high-temperature properties of superalloys. It is generally believed that in the high-temperature creep tests, a higher microstructure stability is expected when the misfit between phases is smaller [33,34]. Due to the expanding of γ lattice, the misfits between γ’’ and γ became larger with increasing solution temperatures. In all heat-treated conditions, the misfit values of δ_γ/γ’’_ were larger than the values of δ_γ/γ’_ because of the different structures between γ’’ (BCT) and γ (FCC) phases. The minimum misfit values were observed for the DA samples, with a δ_γ/γ’’_ and δ_γ/γ’_ of approximately 0.32% and 0.08%, respectively. 

### 3.3. TEM Observation of the SPS IN718 Alloys

**Figure 7:**
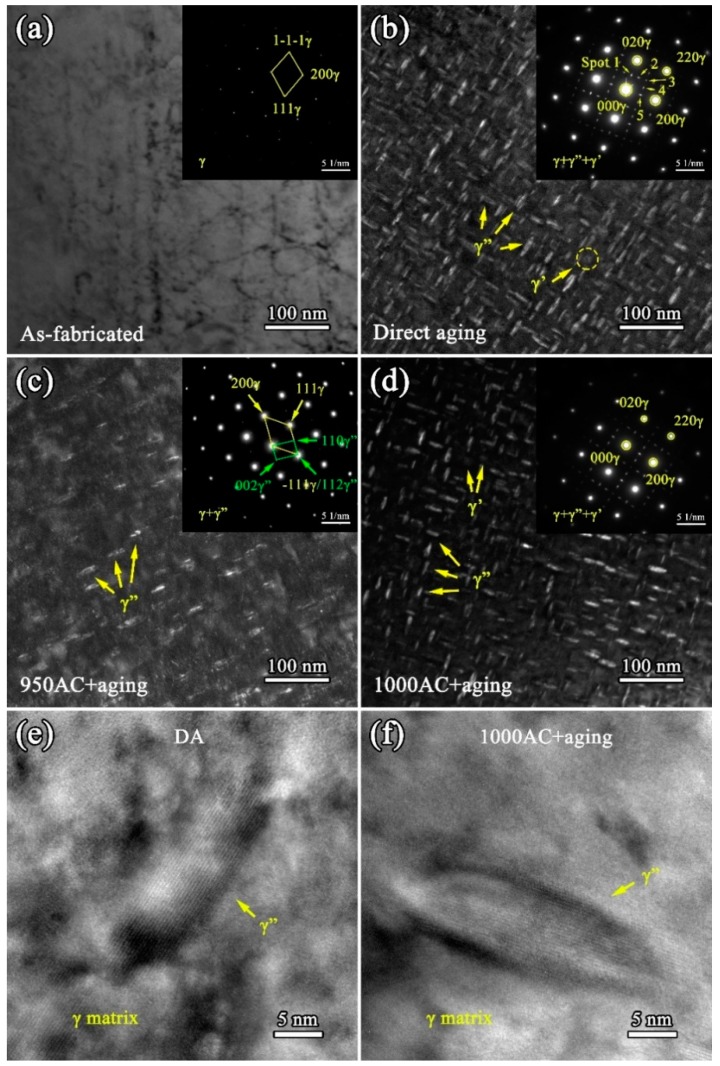
The transmission electron microscopy (TEM) microstructures of the SPS IN718 alloys: (**a**) the bright field (BF) TEM image displays the γ-matrix of the as-fabricated samples (the inset shows the corresponding selected area electron diffraction (SAED) pattern); (**b**), (**c**), and (**d**) show the morphology of the precipitates in the DA, 950AC+aging, and 1000AC+aging samples, respectively (the insets are their corresponding SAED patterns). (**e**,**f**) High resolution (HR)-TEM images of the γ” phases in DA and 1000AC+aging samples.

The TEM results of the SPS IN718 alloys are shown in Figure 7. No obvious γ”/γ’ phase was observed in the bright field TEM image of the as-fabricated samples (see Figure 7a), but some dislocations could be identified in the γ-matrix. The corresponding selected area electron diffraction (SAED) image of the AF sample (see the inset in Figure 7a) only showed the pattern of the γ-matrix. Figure 7b–d shows the morphology of the nano-scale precipitates in the DA, 950AC+aging, and 1000AC+aging samples, respectively. At least three TEM images for each sample were used to calculate the volume fraction of γ” phase by using the image analysis software, ImageJ (Version 1.52J, Bethesda Softworks, Rockville, MD, USA) [35]. The results showed that the volume fractions of γ” phase in DA, 950AC+aging, and 1000AC+aging samples were 23.7%, 5.0%, and 23.2%, respectively. In the DA samples (see Figure 7b), large amounts of disc-shaped γ” phase precipitated along two directions. The length of the γ” phase precipitated in the DA samples was about 25~32 nm, which was nearly the same size as that of γ” in the solution-treated and aged samples. Due to the precipitation of γ’ and the three variants of γ” in the γ matrix during aging treatment, the nano-scale of those precipitates and their structures resulted in the overlapping of SAED patterns at specific orientations. As marked in the SAED patterns of DA samples (see the inset in Figure 7b), spot 1 and 5 could arise from (010) and (100) of [001] γ’ and from (002) of γ” (along the [010] or [100] axis). Spot 2 and 4 belong to the (1/2 1 0) and (1 1/2 0) planes of γ” phase, respectively. The superlattice reflection of (110) (along the [001] axis) for γ’ and γ” have remarkable coincidence at spot 3. The SAED patterns for DA samples were similar to the TEM results of Inconel 718 alloys reported in literature [36,37,38]. 

As shown in Figure 7c, the content of γ” phase in the 950AC+aging samples (5.0 vol%) was much lower than that in the DA samples (23.7 vol%). The SAED patterns of the 950AC+aging samples (see the inset in Figure 7c) failed to recognize the γ’ phase, possibly owing to its low amount. As reported in the Ref. [39,40], γ’ phase is more likely to precipitate when solution-treated at higher temperatures (generally above 1150 °C). The SAED patterns of 950AC+aging samples in this study were similar to the TEM results of the electron beam melted (EBMed) IN718 alloys that were solution-treated at 980 °C [39]. When elevating the solution treatment temperature to 1000 °C, more γ”/γ’ phases precipitated in the alloy compared with 950AC+aging samples. It is because more alloying elements (such as Nb, Al and Ti) were dissolved into the matrix, and a higher supersaturation degree was achieved at 1000 °C, which is beneficial for the precipitating of γ’’/γ’ phases during aging. The SAED patterns of 1000AC+aging (see the inset in Figure 7d) samples were in high accordance with those of the DA samples. Figure 7e,f shows the high-resolution TEM (HR-TEM) images of DA and 1000AC+aging samples, which indicates that the γ’’ phases maintained a perfectly coherent structure with the γ matrix in the SPS IN718 alloy.

### 3.4. Mechanical Properties 

#### 3.4.1. Vickers Hardness 

**Figure 8:**
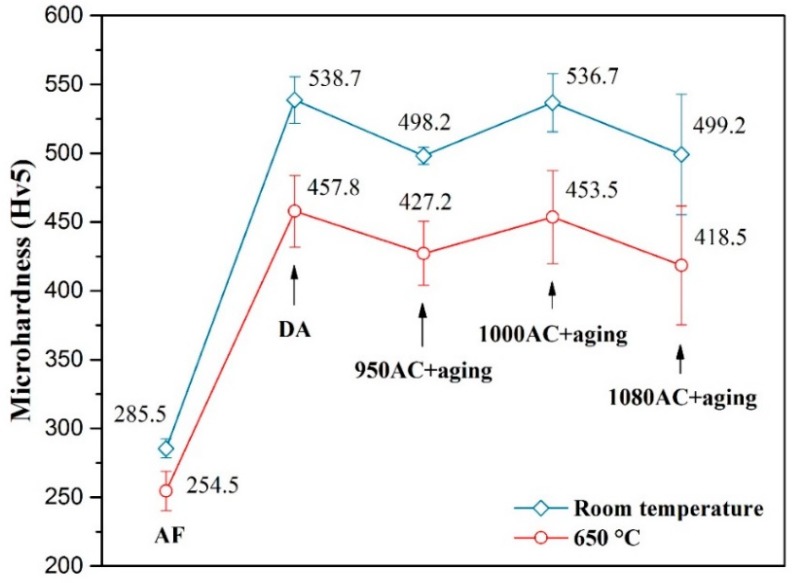
Vickers hardness of the SPS IN718 alloys at room temperature and 650 °C.

In order to evaluate the high-temperature resistance of the SPS-fabricated IN718 alloys, the Vickers hardness tests were conducted at both room temperature (RT) and 650 °C. Figure 8 shows the average hardness values and the errors obtained with at least 10 indentations for each sample. The hardness values of AF samples were much lower than the heat-treated samples, mainly due to the free of γ”/γ’ phases. The maximum hardness value was obtained by DA samples at both RT and 650 °C. Compared with the 950AC+aging samples, the hardness of 1000AC+aging samples increased because of the larger content of γ”/γ’ precipitates. The hardness of 1080AC+aging samples showed an obvious drop mainly caused by the grain coarsening during the solution treatment. The hardness trend at 650 °C was consistent with that at RT. As presented in Figure 8, the micro-hardness decreased for all samples when tested at 650 °C. Such hardness reduction is caused by the thermal softening effect commonly observed in alloys. However, the hardness reduction was within a limited value, indicating that the SPS-fabricated IN718 alloys maintained a good mechanical property at 650 °C.

#### 3.4.2. Compressive Properties

**Figure 9:**
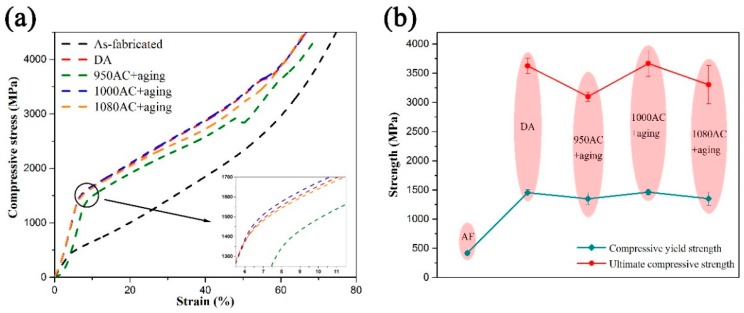
(**a**) The representative compressive stress–strain curves of the SPS IN718 alloys (the inset is the enlarged view of the yielding stage). (**b**) The compressive yield strength and ultimate compressive strength of the IN718 samples.

The representative stress–strain curves of the SPS IN718 alloys are illustrated in Figure 9a, while the 0.2% offset yield strength (σ_0.2_) and the ultimate compressive strength (σ_b_) are presented in Figure 9b. The average values of σ_0.2_, σ_b_, Young’s modulus and densities of the samples are listed in Table 3. It should be noted that no obvious fracture failure was observed after the compressive tests of the SPS IN718 alloys. However, at about 48–57% of the strain, the stress grew slowly or decreased with the increase of strain in the stress–strain curves of the heat-treated samples. It was considered that during this stage, large amounts of cracks initiated and propagated across the samples surface. After this stage, the slopes of stress–strain curves changed obviously due to the work-hardening effect. Therefore, we took the corresponding stress values at the slowly-growing (or decreasing) stage as the ultimate compressive strength of the samples. 

As shown in Figure 9, the yield strength of the as-fabricated samples was much lower than the heat-treated samples, which was consistent with the micro-hardness test results. After heat treatment, the compressive yield strength of the alloy improved significantly mainly due to the precipitating of γ”/γ’ strengthening phases. The 1000AC+aging samples exhibited the highest 0.2% offset yield strength (1463 MPa) and ultimate compressive strength (3666 MPa). However, the 950AC+aging samples (heat-treated by the standard route) showed the minimum compressive properties in the heat-treated samples. Compared with the 950AC+aging samples, the σ_0.2_ and σ_b_ values of 1000AC+aging samples increased by ≈9% and ≈18%, while the σ_0.2_ and σ_b_ values of DA samples were ≈8% and ≈17% higher. The compressive properties of 1080AC+aging samples were lower than the DA and 1000AC+aging samples, which could be ascribed to the larger grain sizes of 1080AC+aging samples. 

## 4. Discussion

### 4.1. The Precipitating Mechanisms of γ”/γ’ in the DA Condition

**Figure 10:**
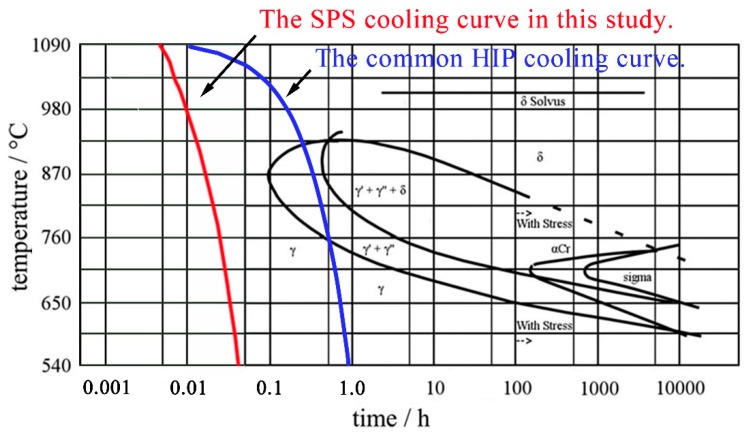
The time-temperature-transformation (TTT) diagram of IN718 alloy [41] with the SPS and hot-isostatic-pressing (HIP) cooling curves.

As can be observed from Figure 7b, the γ”/γ’ phases precipitated extensively in the DA samples. However, the precipitating mechanism of γ”/γ’ phases in DA samples was different from that of the solution-treated samples. In the as-fabricated samples, γ”/γ’ phases did not precipitate after SPS, as can be observed from Figure 7a. However, large numbers of γ”/γ’ phases precipitated after direct aging treatment. This phenomenon can be explained by comparing the SPS cooling curve in this study with the IN718 TTT diagram [41]. As can be seen from Figure 10, the SPS cooling was very rapid and the cooling curve did not pass through the γ”/γ’ precipitation zones. When SPS sintering at 1200 °C, alloying elements were dissolved into the γ matrix. During the fast cooling period of SPS, the dissolved elements did not have enough time to diffuse evenly or precipitate as secondary phases in the samples, and the AF samples obtained a supersaturated solid solution state after SPS. The following direct aging treatment activated the precipitation of γ” and γ’ phases. Combined with TEM results, the amount of γ” and γ’ in DA samples was considerably large. This is the main reason that high hardness and compressive strength were acquired by DA samples. By comparison with the DA samples, the solution-treated and aged samples were subjected to a second solution treatment, and the solubility of the alloying elements mainly depended on the solution treatment temperature. After air cooling, samples acquired the supersaturated solid solution state, which enabled the precipitating of strengthening phases in the following double-aging process. 

The blue curve in Figure 10 shows the frequently used hot-isostatic-pressing (HIP) cooling rate of 10 °C/min (close to the cooling speed limit of HIP). It can be seen that the HIP cooling curve goes straight into the γ”/γ’ zones due to the lower cooling rate than SPS. As reported in the literature [18,42], for the HIP-fabricated IN718 alloy, γ”/γ’ phases already precipitated in the samples before heat treatment. The precipitation of the γ”/γ’ phases in as-fabricated samples led to the release of the matrix supersaturation. When directly aging the HIPed IN718, only the pre-existing γ”/γ’ precipitations grew and coarsened; new γ”/γ’ could not precipitate due to the low driving force for their nucleation. Similar situations can also be found in the IN718 alloys prepared by electron beam melting (EBM) [12,43] and selective laser melting (SLM) [14] methods. For the HIPed IN718, the mechanical properties of DA samples are inferior to the solution-treated samples. In this study, the hardness of SPS-fabricated IN718 in DA condition was higher than the solution-treated samples. In addition, the compressive properties of DA samples were comparable to the best one in the solution-treated and aged samples. This indicates that DA is the optimized heat treat route for SPS-fabricated IN718 alloys because it ensures the excellent mechanical properties and reduces the heat treatment steps.

### 4.2. The Relations of the Microstructure and Mechanical Properties

After heat treatment, the hardness and compressive properties increased significantly, mainly because of the precipitation of γ” and γ’ phases during aging. However, the precipitation of δ and carbide phases can greatly influence the precipitation behavior of γ”/γ’ phases and thus also the mechanical properties. It should be noted that the δ-Ni_3_Nb phase shares the same elemental compositions with γ” phase, and γ” phase can transform into δ during heat treatment [8,9,29]. At a given concentration of Nb, the precipitation of δ phase will consume Nb atoms, resulting in the decrease of the γ” content. As reported in the literature [13], large numbers of δ phase precipitated in the alloy are detrimental for sample mechanical properties because it competes Nb elements with γ” phase. Meanwhile, appropriate amounts of δ at grain boundaries can restrain the grain growth, which is beneficial for enhancing the mechanical properties [11,12]. For the 950AC+aging samples, the content of δ phases was large (see Figure 4) and their sizes were larger than those in the 1000AC+aging samples. This is because the precipitation rate for δ phase is the highest at around 900 °C [44]. Owing to the considerable quantities of Nb elements consumed by the δ phase, less γ” phase precipitated in 950AC+aging samples (see Figure 7c), resulting in the inferior mechanical properties. With the aim of promoting the formation of γ” and limiting the amount of δ phase, we elevated the solution annealing temperature to 1000 °C. As a result, both the amount and length of δ phase were reduced in 1000AC+aging samples (see Figure 4). The γ” phases in the 1000AC+aging samples were obviously denser than those in the 950AC+aging samples (see Figure 7c,d), which directly enhanced the sample hardness and compressive strength. Moreover, when solution-treated at 1000 °C, more alloying elements could diffuse into the matrix lattice, which is thermo-dynamically favorable for precipitating γ”/γ’ phases. In the 1080AC+aging samples, no needle-shaped δ phase could be found because 1080 °C exceeds the solvus of δ phase (around 1050 °C [11]). Consequently, grain growth could not be restricted by the grain boundary δ phases. Therefore, the coarsening of grains is possibly the main reason for the inferior mechanical performance of 1080AC+aging samples. The DA regime activates the precipitation of large amounts of γ”/γ’ phases and avoids the grain growth caused by solution annealing. Therefore, a novel mechanical property can be achieved by DA, and it seems that the solution treatment is abundant for the SPS-fabricated IN718 alloy.

In the present work, both the sintering and heat treatment temperatures do not exceed the solvus of NbC (around 1250 °C, as mentioned above); thus, blocky carbide phases could not be removed from the IN718 samples (see Figure 4). Generally, the existence of carbides is harmful for the mechanical properties of the sample because carbides can easily cause stress concentration near the tips and become the crack initiation site [18]. However, it is also reported that the formation of carbides at grain boundaries is preferred because they can improve the grain boundary strength by inhibiting the movement of dislocations [13]. Moreover, carbides can restrain the grain growth by the pinning effect, which is beneficial for improving sample mechanical properties [45]. According to existing literature, the role of carbides has proved controversial. Besides, the low carbon content in IN718 suggests that only a small number of carbide phases can be formed in the samples. Therefore, it is considered that the existence of carbide phase has a minor influence on the sample mechanical properties. 

### 4.3. Microstructural Evolution during SPS and Heat Treatment

**Figure 11:**
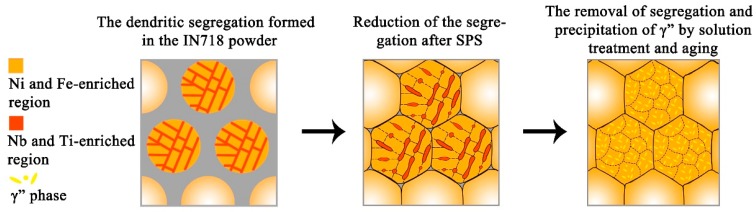
Schematic illustration of the microstructural evolution of SPS IN718 alloy during sintering and heat treatment.

As mentioned above, in the IN718 powder, the segregation of Nb and Ti caused the dendritic microstructure (see Figure 2a,g–i). The microstructure of the as-fabricated samples showed the dendritic segregation with discontinuous outlines (see Figure 3a). The EDS element distribution map (Figure 3) showed that element types (Nb and Ti) segregated in the interdendritic regions of the as-fabricated samples were the same as those segregated in the IN718 powder. Moreover, the average secondary dendrite arm spaces (SDAS) of the powder and the SPS-fabricated IN718 alloy were almost the same (1.64 μm and 1.66 μm, respectively). As reported in Ref. [13,46,47,48,49], the micro-segregation of Nb, Mo, and Ti elements in the interdendritic region is very common in the cast, wrought, and P/M IN718. After solution treatment and aging, the segregated elements diffused evenly into the sample matrix. Based on the sample characterization results and the discussions above, the microstructural evolution of the SPS IN718 is schematically illustrated in Figure 11. The first image of the schematics shows that Nb and Ti elements initially segregated in the interdendritic region of the raw powder due to the solidification partition coefficient difference of the alloying elements. Then, the element segregation was reduced by SPS sintering the powder compact at 1200 °C for 10 min. However, due to the SPS soaking time being too short, the dendritic segregation was not totally eliminated and became discontinuous in the as-fabricated samples. Lastly, the homogenization effect of solution treatment promoted the alloy elements to diffuse evenly into the matrix. The final microstructure of the solution-treated and aged samples mainly contains the γ-matrix and γ” phases.

## 5. Conclusions

In this study, the SPS technology was employed to prepare the IN718 alloy, and the effects of post heat treatment on the microstructural evolution, phase transformation and mechanical properties of SPS IN718 alloys were studied. The conclusions drawn are as follows:

1. Direct aging promotes the full precipitation of γ”/γ’ phases and reduces the heat treatment steps, which is considered as the optimal heat treatment regime for the SPS-fabricated IN718 alloy. Compared with the samples heat-treated by the standard route of as-cast IN718, both hardness and compressive properties of the DA samples showed obvious enhancement.
2. The microstructural evolution of the SPS IN718 was ascribed to the reduction of elemental segregation during SPS and heat treatment. The post-sintering heat treatment tailored the phase compositions in the alloy, and thus mechanical properties were enhanced by this method. The SPS IN718 alloys exhibited excellent high-temperature resistance at 650 °C.
3. In addition, the method provided herein is an efficient and economic process for enhancing the mechanical properties of the SPS IN718 alloy, which was achieved by the direct aging regime. The DA samples have comparable strength with the best one of the solution-treated and aged samples. The approach can provide potential guidance for improving mechanical properties in other precipitation strengthening alloys by excluding the energy-intensive solution treatment.

## Figures and Tables

**Table 1:** The chemical composition of the plasma rotation electrode process (PREP)-processed Inconel 718 powder.

Element	Ni	Cr	Nb	Mo	Ti	Al	Co	Mn	Si	Cu	C	Fe
Wt.%	54.28	17.97	5.12	3.00	0.98	0.57	0.21	0.09	0.077	0.069	0.024	Bal.

**Table 2:** Lattice parameters (nm), and misfit values δ (%) of γ, γ’, and γ’’ phases calculated on the basis of peak fitting of the XRD data.

Heat Treatment	Direct aging	950AC+aging	1000AC+aging	1080AC+aging
a_γ_	0.35907	0.36019	0.36163	0.36166
a_γ’_	0.35904	0.35899	0.36097	0.36044
a_γ’’_	0.35761	0.35874	0.35877	0.35882
c_γ’’_	0.71648	0.71834	0.71983	0.71821
δ_γ/γ’_	0.08412	0.33384	0.18254	0.33868
δ_γ/γ’’_	0.32121	0.34511	0.63827	0.75209

**Table 3:** Mechanical properties, densities and relative densities of the SPS IN718 alloys after different heat treatments.

Samples	σ_0.2_ (MPa)	σ_b_ (MPa)	Young’s Modulus (GPa)	Density (g·cm^−3^)	Relative Density (%)
As-fabricated	419	-	13.83	7.66	93
DA	1454	3626	27.50	8.07	98
950AC+aging	1349	3100	24.80	8.15	99
1000AC+aging	1463	3666	26.55	8.16	99
1080AC+aging	1350	3303	26.18	8.20	99
